# All that hemolyzes with complement is not cold agglutinin syndrome

**DOI:** 10.1590/1984-0462/2024/42/2023144

**Published:** 2023-12-18

**Authors:** Jeremy Jacobs, Garrett Booth, Brian David Adkins

**Affiliations:** a Mayo Clinic, Department of Laboratory Medicine and Pathology, Rochester, MN, USA.; b Vanderbilt University Medical Center, Department of Pathology, Microbiology, and Immunology, Nashville, TN, USA.; c University of Texas Southwestern Medical Center, Department of Pathology, Dallas, TX, USA.

Sion et al. recently described a 2-year-old child with apparent autoimmune hemolysis in the setting of human adenovirus infection.^1^ Per the authors, the patient developed signs of intravascular hemolysis with hemoglobinuria and a precipitous hemoglobin drop from 11.6 to 2.6 g/dL. The authors appropriately performed a direct antiglobulin test (DAT), which was positive for complement (C3d), and an indirect antiglobulin test (IAT) at 4°C, which was also positive, though further details are not provided. The authors ultimately attributed this to cold agglutinin (CA) syndrome (CAS). Unfortunately, immunohematological evaluation is insufficient to definitively conclude a diagnosis of CAS, and most likely may be explained by paroxysmal cold hemoglobinuria (PCH).

CAS is characterized by autoantibodies, typically of the IgM isotype, which are optimally reactive at 4°C. These autoantibodies bind complement, and although complement is capable of proceeding through the membrane attack complex (MAC) with subsequent intravascular hemolysis, intravascular hemolysis is significantly rarer than extravascular hemolysis via phagocytosis of C3b-opsonized red blood cells (RBCs) by Kupfer cells in the liver.^2^ Diagnosis of CAS requires demonstration of CAs at 4°C, which almost always display a titer ³64, often in the range of 100s to 1,000s.^3^ However, thermal amplitude (TA), or the highest temperature at which the autoantibody is capable of mediating hemolysis, is more indicative of its potential significance, with clinically-significant CAs displaying TA ³28-30°C.^3^ Unfortunately, the authors either did not test for, or at least report, the titer and TA of the implicated CA in this particular case. Moreover, additional tests to support a diagnosis of CAS, including a 4°C and a 37°C autocontrol and IAT and antibody specificity, were not reported. A drug history was not provided, and this is specifically relevant as antibiotics are often prescribed for upper respiratory infections and can present with drug-induced anemia with positive DAT, which is often found with cephalosporins.^4^

Conversely, PCH is characterized by the Donath-Landsteiner (DL) antibody, which is almost invariably of the IgG isotype and directed against the P antigen on RBCs. This antibody was historically identified in patients with tertiary syphilis, but following the advent of effective antitreponemal therapy, it is now typically observed in the pediatric population following upper respiratory infection, with multiple cases described in association with adenovirus.^5^ The presence of systemic symptoms, marked anemia, and intravascular hemolysis with hemoglobinuria are all consistent with the findings of PCH.^5^ Furthermore, the DAT positive for C3 further supports this diagnosis; to confirm, a DL test or, at the very least, a CA titer and TA would have been informative. The DL antibody characteristically binds to RBCs in the cold (or in peripheral circulation) and fixes the initial stages of complement on the surface of RBCs. As the RBCs return to the core circulation, the antibody dissociates, and the complement cascade proceeds through the formation of the MAC and frequent intravascular hemolysis.

In addition to the diagnostic dilemma posed by this case, particularly in the absence of the requisite immunohematologic evaluation, the authors’ suggestion that plasmapheresis is helpful as a temporizing measure warrants addressing. While the American Society for Apheresis 2023 guideline supports therapeutic plasma exchange (TPE) as second- or third-line therapy in the setting of fulminant hemolysis unresponsive to initial therapies in patients with cold autoimmune hemolytic anemia,^6^ TPE is *not effective* at removing “sensitized RBCs”, despite the authors’ suggestion. Instead, TPE simply separates the patient’s blood into its components, and given that approximately 60% of IgM is intravascular, removal and exchange of plasma will temporarily deplete the pathogenic IgM autoantibody. Moreover, while historically authors may have suggested that RBC units should be washed to reduce their plasma content, and therefore the complement, contemporary RBC products contain 15–30 mL of plasma, and no study has shown that this minimal plasma content contains sufficient complement to induce or prolong complement-mediated hemolysis. Thus, it is our professional opinion that washing, which can reduce the cellular content of blood products by as much as 30%, or otherwise ‘plasma-reducing’, RBC units are both ineffective and a waste of resources.

In summary, we appreciate the report by Sion et al.^1^ describing a young child with complement-mediated hemolytic anemia; however, we caution readers from concluding this case represents “the first description of a child that had no known underlying malignancy or infectious disease typically associated with CAS” as suggested by the authors, given the lack of complete immunohematologic testing. Secondly, we maintain that there are currently more effective therapies than TPE in patients developing cold agglutinin disease, with several additional drugs, including upstream complement inhibitors such as sutimlimab-jome, on the horizon.^7^ Finally, we suggest, in our collective expertise, that modification to reduce the plasma content of RBCs is not required for this patient population, as this manipulation may induce more harm than benefit.
