# Supplementary material for: All that hemolyzes with complement is not cold agglutinin syndrome
Source: Rev Paul Pediatr. 2023 Dec 18;42:e2023144. doi: 10.1590/1984-0462/2024/42/2023144 (PMC10741275; doi:10.1590/1984-0462/2024/42/2023144)
Supplement: Related texts

# Reply to letter to the editor

**Authors:** Julia Loureiro Sion, Angelica Lucía Hidalgo Flores, Regina Aparecida Cardoso, Marlene Pereira Garanito

**Affiliations:** a Universidade de São Paulo, São Paulo, SP, Brazil.

Dear Editor of Revista Paulista de Pediatria (RPPed),

We thank you for the letter in which our article “Human adenovirus associated with severe cold agglutinin syndrome: a rare complication in Pediatrics” was appreciated. We will respond to all considerations within our limitations.

In their letter, the specialists shared concerns about the immunohematological evaluation and Cold Agglutinin Syndrome (CAS) diagnosis. Regarding the immunohematological studies, our detailed results were: group O, Rh positive, polyspecific direct antiglobulin test (DAT) positive just for C3d, with strong agglutination (4+). The monospecific DAT was negative for IgG. Indirect antiglobulin test (IAT) performed at 4, 22, and 37°C showed cold agglutinin antibodies reacting at 4°C, and the auto control was positive at 4°C and negative at 37°C. We did not test the antibody titers because the technique was not available in our laboratory. It is important to consider that, classically, autoimmune hemolytic anemia (AIHA) is characterized by the presence of a cold agglutinin (CA) titer of 64 or higher at 4°C. However, according to recent literature, the titer level is less concordant with disease activity once hemolysis can occur with levels as low as <1:32.^1,2^

Regarding the drug history, it was not our intention to omit this data. This patient did not receive any antibiotics prior to the anemia, ruling out the diagnosis of drug-induced anemia.

We agree that therapeutic plasma exchange (TPE) is not effective at removing “sensitized red blood cells”. This procedure just temporarily depletes the pathogenic IgM autoantibody and may be helpful as a temporary measure in severe and selected cases in the acute phase of pediatric CAS secondary to infection. According to the “Guidelines on the Use of Therapeutic Apheresis in Clinical Practice – Evidence-Based Approach from the Writing Committee of the American Society for Apheresis: The Ninth Special Issue, 2023”, in severe Cold Agglutinin, TPE is category 2 (Disorders for which apheresis is accepted as second-line therapy, either as a stand-alone treatment or in conjunction with other modes of treatment).^3^

Regarding more effective therapies than TPE currently available for patients developing cold agglutinin disease (CAD), with several additional drugs, including upstream complement inhibitors such as sutimlimab-jome, to our knowledge, no evidence-based therapy is available for CAS secondary to infection in pediatric patients. Prospective trials or well-designed retrospective series have not been published. As discussed in our report, according to Voulgaridou and Kalfa^4^ and Kin and Despotovic,^5^ steroid therapy or other immunosuppressive medications are not indicated. The use of Rituximab is cited in rare case reports.^6^ For these reasons, plasmapheresis may be helpful as a temporizing measure until resolution of infection. CAD is an autoimmune hemolytic anemia associated with a clonal lymphoproliferative disorder. We agree that more effective therapies than TPE currently exist for adults with the disease. For example, Sutimlimab and Bendamustine.^7^ We did not discuss CAD once it was not the objective of our report and these therapies are not available for pediatric patients.

We thank the specialists for taking the time to comment on our recently published case report.

## References

[1] Swiecicki PL, Hegerova LT, Gertz MA (2013). Cold agglutinin disease. Blood.

[2] Berentsen S (2020). New insights in the pathogenesis and therapy of cold agglutinin-mediated autoimmune hemolytic anemia. Front Immunol.

[3] Connelly-Smith L, Alquist CR, Aqui NA, Hofmann JC, Klingel R, Onwuemene OA (2023). Guidelines on the use of therapeutic apheresis in clinical practice – evidence-based approach from the writing committee of the American Society for Apheresis: the ninth special issue. J Clin Apher.

[4] Voulgaridou A, Kalfa TA (2021). Autoimmune hemolytic anemia in the pediatric setting. J Clin Med.

[5] Kim TO, Despotovic JM (2019). Primary and secondary immune cytopenias: evaluation and treatment approach in children. Hematol Oncol Clin North Am.

[6] Berentsen S, Randen U, Oksman M, Birgens H, Tvedt TH, Dalgaard J (2017). Bendamustine plus rituximab for chronic cold agglutinin disease: results of a Nordic prospective multicenter trial. Blood.

[7] Enjaymo: Sutimlimab-jome [homepage on the Internet] (2023). Bioverativ Therapeutics Inc., Waltham, MA 02451. A SANOFI COMPANY. US License No. 2078.
